# Durability of clinical and immunologic responses to extended low-dose interleukin-2 therapy in patients with refractory chronic graft-versus-host disease

**DOI:** 10.3389/fimmu.2022.954966

**Published:** 2022-09-14

**Authors:** Veronica Donato, Haesook T. Kim, Peter Stowe, Carol G. Reynolds, Jerome Ritz, John Koreth, Jennifer S. Whangbo

**Affiliations:** ^1^Division of Hematologic Malignancies, Dana-Farber Cancer Institute, Boston, MA, United States; ^2^Master of Medical Sciences in Clinical Investigation Program, Harvard Medical School, Boston, MA, United States; ^3^Department of Data Sciences, Dana-Farber Cancer Institute, Boston, MA, United States; ^4^Harvard School of Public Health, Boston, MA, United States; ^5^Harvard Medical School, Boston, MA, United States; ^6^Division of Hematology-Oncology, Boston Children’s Hospital, Boston, MA, United States; ^7^Department of Pediatric Oncology, Dana-Farber Cancer Institute, Boston, MA, United States

**Keywords:** regulatory T cells, low-dose interleukin-2, chronic graft vs. host disease, allogeneic stem cell transplantation, steroid refractory graft versus host disease

## Abstract

Chronic graft-versus-host disease (cGVHD) remains a frequent cause of non-relapse morbidity and mortality after allogeneic hematopoietic stem cell transplantation. In our single center trials of low-dose interleukin-2 (LD IL-2), the immunomodulatory properties of regulatory T cells (Tregs) have been harnessed to treat steroid-refractory cGVHD (SR-cGVHD) safely and effectively in adults and children. In these trials, 50-60% of patients showed clinical improvement of their cGVHD manifestations with partial responses at the primary response endpoint of 8-12 weeks. Many patients continued extended duration LD IL-2 therapy and achieved deeper clinical responses, including some complete responses. However, the durability of the clinical and immunologic improvement following IL-2 discontinuation has not been reported previously. We examined 20 adult and 2 pediatric patients who received extended duration LD IL-2 for a median of 103 weeks (range, 21-258) and had stable improvement or resolution of their cGVHD symptoms before discontinuing LD IL-2 therapy. The median follow-up after stopping IL-2 was 203 weeks (range 92-599). During this time, 16 patients (73%) were able to wean off all systemic immunosuppression without disease flare or progression. Among 13 patients with available immune cell data, the median fold change in absolute Treg count was 0.58 between 1 to 10 weeks after stopping IL-2 whereas CD4+ conventional T-cell (Tcon) and CD8+ T-cell numbers remained stable. Despite a decline in Treg numbers after IL-2 discontinuation, Treg numbers remained above the pre-treatment baseline. In addition, many patients had sustained clinical improvement after stopping IL-2, suggesting that extended IL-2 therapy can lead to immune tolerance.

## Introduction

Chronic graft-versus-host disease (cGVHD) is the leading cause of non-relapse morbidity and mortality following conventional allogeneic hematopoietic stem cell transplantation (HSCT), occurring in 60-70% of adults and 20-50% of children surviving greater than 100 days post-transplant (1–3). Systemic steroids are the first-line therapy for cGVHD but have limited efficacy and considerable toxicity. Although there are now 3 Food and Drug Administration (FDA)-approved second-line therapies for steroid-refractory (SR)-cGVHD (4), many patients require prolonged treatment courses with multiple lines of therapy. Patients with cGVHD have impaired reconstitution of CD4+CD25+CD127-FOXP3+ regulatory T cells (Tregs), which normally comprise 5-10% of the circulating CD4+ T cell population and function to control inappropriate auto- and allo-reactive immune responses (5–9). Based on the observation that patients with cGVHD had lower Treg numbers, it was hypothesized that preferential augmentation of Treg may be helpful in the control of cGVHD. At our institution, we have evaluated *in vivo* Treg expansion with low-dose interleukin-2 (LD IL-2) treatment, which promotes thymic differentiation and peripheral proliferation, survival, and function of Treg (10–12). Multiple clinical trials have established that daily subcutaneous (SC) LD IL-2 administered at a dose of 1x10^6^ IU/m^2^/day is safe and well-tolerated for prolonged periods in both adults and children. Importantly, this regimen induced preferential Treg expansion with objective clinical responses in 50-60% of adults and 80% of children with SR-cGVHD (13–16).

Clinical responses to LD IL-2 were evident after 8 to 12 weeks of daily injections although no patients had complete responses during this time period. However, many patients with clinical benefit (partial responses or stable disease with minor response) on these trials continued extended duration LD IL-2 therapy and had further improvement of their cGVHD manifestations, including some complete responses. The optimal treatment course of LD IL-2 and durability of clinical response are unknown. Patients with continued improvement of their cGVHD were often reluctant to stop treatment due to concern for flaring of their symptoms and preferred to continue daily LD IL-2 injections until they weaned off all other immunosuppression or had plateauing of their response. Here we examined the clinical course after LD IL-2 discontinuation in 22 patients who received extended therapy for a median of 103 weeks (range, 21-258) and had stabilized or plateaued clinical responses. We excluded patients who discontinued LD IL-2 due to cGVHD progression or malignancy relapse. For the first time, we describe long term clinical and immunologic follow up after stopping LD IL-2 therapy. We found that the clinical improvement with LD IL-2 was durable as 16 of these 22 patients were able to wean off all cGVHD-directed immunosuppression without recurrence or worsening of cGVHD during a median follow up period of 203 weeks (range, 92-599).

## Methods

### Patients and clinical trials

We performed a retrospective chart review of 22 patients from five single-center phase I or II clinical trials conducted at the Dana-Farber Cancer Institute from 2008 through 2017 (NCT# 00529035, 01366092, 01937468, 02318082, 02340676). These trials investigated the safety and efficacy of *in vivo* LD IL-2 therapy for treatment of SR-cGVHD. The initial treatment period was 12 weeks for NCT01366092 and 8 weeks for the other 4 trials. Upon completion of the initial treatment period and clinical response assessment, patients with partial response (PR), stable disease (SD), or minor response (MR) could continue daily LD IL-2 therapy indefinitely. For this report, we selected patients who discontinued LD IL-2 after having stable disease or continued improvement of their cGVHD manifestations during extended duration therapy. Patients who discontinued LD IL-2 due to adverse events (including malignant relapse, secondary malignancy, and death), cGVHD progression, or addition of a new line of therapy for cGVHD were excluded with the exception of one patient with CLL relapse (C18) who was treated with ibrutinib only. All five trials were approved by the Dana-Farber/Harvard Cancer Center Institutional Review Board. Prior written informed consent was obtained per the Declaration of Helsinki.

### Flow cytometric analysis of lymphocyte subsets

CD4+ Treg were defined as CD3+CD4+CD25^med-high^CD127^low^; CD4+ Tcon as CD3+CD4+CD25^neg-low^CD127^med-high^; and CD8+ T cells as CD3+CD4-CD8+. Subsets within each population were defined as: naïve T cells (CD45RO-CD62L+), central memory/CM (CD45RO+CD62L+), effector memory/EM (CD45RO+CD62L-), and CD8 effector memory RA/TEMRA T cells (CD8+CD45RO-CD62L-). Labeled cells were acquired in a FACSCanto II flow cytometer (BDBiosciences) and analyzed using FlowJo software (Tree Star). Gating and analysis strategies have been described previously (9, 14).

## Results

### Patient characteristics

From 2007 through 2017, one hundred twenty-three adult patients and eleven pediatric patients with SR-cGVHD were enrolled on five IL-2 clinical trials at our institution (17). Fourteen patients were not evaluable for response and were excluded from further analyses. Seventy patients (59 adults, 11 pediatric) with clinical benefit continued extended-duration LD IL-2, and 50% of these patients (30 adults, 5 pediatric) had continued improvement at various organ sites during extended therapy. To examine the durability of cGVHD improvement in the absence of continuous exposure to LD IL-2, we selected patients who discontinued LD IL-2 after achieving maximal clinical improvement of their cGVHD manifestations or stable disease with the ability to wean off other immunosuppressive medications. Baseline clinical and transplant characteristics of the 22 patients included in this study are presented in **Table 1**. The median age at study enrollment was 48 years (range, 2-76 years), 55% were male, median time from cGVHD onset to study enrollment was 41 weeks (range, 10-241); median number of prior therapies was 2 (range, 1-7), and median number of sites involved was 3 (range, 2-6). At enrollment, all 22 patients were concurrently taking prednisone at a median dose of 22.5 mg daily (range, 5-50).

**Table 1:** Baseline patient characteristics.

	N	%
Total	22	100
Age at enrollment		
median (range)	48 (2, 76)	
Time from BMT to Study		
median (range) in weeks	107 (39, 310)	
Time from cGVHD onset to Study		
median (range) in months	41 (10, 241)	
No. of cGVHD sites involved		
median (range)	3 (2, 6)	
No. of prior therapies including steroid		
median (range)	2 (1, 7)	
Daily prednisone dose		
median (range), mg	22.5 (5, 50)	
Prior Grade I-IV acute GVHD	9	41
Patient Sex		
Male	10	45
Female	12	55
Diagnosis		
AML	6	27
ALL	3	14
CLL/SLL/PLL	5	23
MDS	4	18
NHL	2	9
CML	1	5
Anemia/Red Cell Disorder	1	5
Conditioning Intensity		
MAC	12	55
RIC	10	45
HLA type (A, B, C, DRB1)		
Matched Unrelated	11	50
Matched Related	8	36
Mismatched Unrelated	3	14
Cell Source		
Bone marrow	4	18
PBSC	18	82

### IL-2 treatment course

Details of each patient’s clinical course are summarized in **Table 2**. At the response assessment following the initial 8-to-12-week IL-2 treatment course, 12 patients (55%) had PR, 9 patients (41%) had SD or SD/MR, and 1 patient (5%) had a mixed response. During the extended treatment course, 2 patients with previous PR achieved CR and 3 patients with SD, SD/MR, or mixed response at the initial response assessment improved and met objective PR criteria. Thus, the best overall response rate for this cohort was 68% (13 PR, 2 CR). Patients discontinued extended LD IL-2 therapy after a median duration of 103 weeks (range, 21-258). The most common reason for discontinuation of LD IL-2 was due to plateaued response or stabilized disease in 18 patients (82%). Along with the lack of further improvement, several patients also cited intolerable side effects of LD IL-2 or difficulty with returning to clinic every 2 weeks to pick up their IL-2 supply. One patient stopped IL-2 after achieving CR and one patient stopped due to difficulty with finding injection sites due to the formation of inflammatory skin nodules at prior injection sites. Two patients did not cite specific reasons for wanting to discontinue LD IL-2 therapy.

**Table 2:** Clinical course during and after low-dose IL-2 therapy.

Case no.	Pt sex	Age	cGVHD dx to study entry (Wks)	No. prior lines tx	Sites involved and cGVHD severity at study entry	LD IL-2 course (Wks)	Response at Week 8 or Week 12	Best overall response	Reason for d/c IL-2	IL-2 stop to last f/u (Wks)	cGVHD status at last f/u	Systemic IS at last f/u
1	M	48	161	1	Skin, JMF; severe	74	PR	PR	Plateaued response	599	Improving subcutaneous fibrosis	None
2	M	65	72	3	Skin, JMF; moderate	132	PR	PR	Plateaued response, difficulty finding injection sites	191	Stable thickened skin	sirolimus, LD prednisone
3	F	22	70	3	Skin, eyes, JMF; severe	86	PR	PR	Plateaued response	204	No active disease; hyperpigmented skin	None
4	F	46	13	2	Mouth, eyes, liver, GU; moderate	258	PR	CR (liver); PR (eyes and mouth)	Stabilized disease, mild fatigue and difficulty picking up IL-2 supply	234	Mild oral and ocular GVHD	None
5	M	29	27	1	Skin, lung, liver, GI, JMF; moderate	244	PR	PR	Plateaued response	244	No active disease	None
6	F	50	71	2	Skin, mouth, JMF; severe	138	PR	PR	Stabilized disease, difficulty picking up IL-2 supply	190	Stable minimal skin cGVHD	None
7	M	61	17	2	Skin, eyes, JMF; moderate	24	SD/MR	SD/MR	Difficulty with daily injections	442	Minimal cGVHD of the eyes, well-controlled	None
8	F	22	50	2	Skin, mouth, eyes, lung, liver, GU; moderate	102	PR	PR	Patient decision	227*	Stable respiratory status on trach/vent^#^, active skin cGVHD	Tacrolimus, prednisone
9	M	68	226	1	Mouth, eyes, lung; moderate	57	SD/MR	SD/MR	Plateaued response	383	No active disease	None
10	F	70	32	2	Skin, mouth, eyes, lung, liver, JMF; moderate	94	SD/MR	SD/MR	Plateaued response, side effects (anorexia and fatigue)	343	Continued sclerodermatous skin	Ruxolitinib, LD prednisone
11	F	45	10	1	Skin, mouth, eyes, liver, GU; moderate	21	PR	PR	Plateaued response	432	Persistent dry eyes, dry mouth; mild skin involvement	Ruxolitinib
12	F	34	34	2	Skin, eyes, lung, JMF; moderate	80	SD/MR	SD/MR	Stabilized disease, difficulty picking up IL-2 supply	92	No active disease	None
13	M	48	116	2	Skin, mouth, eyes, JMF; moderate	84	SD/MR	SD/MR	Plateaued response	141	Persistent mild skin and JMF involvement	None
14	M	44	241	7	Skin, JMF; severe	104	SD/MR	SD/MR	Plateaued response	206	Persistent severe cGVHD of skin and JMF	Sirolimus, ibrutinib
15	F	59	29	2	Skin, JMF; severe	106	PR	CR	Resolved cGVHD	148	No residual disease	None
16	M	57	26	3	Skin, mouth, liver; moderate	83	PR	PR	Patient decision	201	Residual scleroderma	None
17	F	59	30	3	Skin, eyes, lung, JMF; moderate	177	SD/MR	PR	Plateaued response, side effects	119	Some residual tightness of skin	None
18	F	76	128	3	Skin, eyes, JMF; severe	121	SD/MR	SD/MR	Plateaued response; CLL relapse	196	Residual scleroderma	Ibrutinib
19	F	65	41	2	Skin, lung, JMF; moderate	57	SD	PR	Plateaued response	238	No active disease	None
20	F	21	121	2	Mouth, eyes, GI; moderate	123	Mixed	PR	Plateaued response	137	Persistent eye involvement	None
21	M	12	41	2	Skin, mouth, lung; moderate	141	PR	CR	Plateaued response	100	No active disease; hyperpigmented skin	None
22	M	2	16	2	Skin, mouth, liver, JMF; moderate	139	PR	PR	Plateaued response	101	No active disease; residual hyperpigmented skin and joint contractures	None

*Patient died of multisystem organ failure after a prolonged hospitalization for appendicitis.

^#^ Patient had progression of lung cGVHD after an infection and underwent tracheostomy with ventilator dependence.

cGVHD, chronic graft-versus-host disease; CLL, chronic lymphocytic leukemia; CR, complete response; Dx, diagnosis; D/c, discontinue; F/u, follow up; GI, gastrointestinal tract; GU, genitourinary tract; IS, immunosuppression; JMF, joint/muscle/fascia; LD, low dose; No., number; PR, partial response; Pt, patient; SD/MR, stable disease with minor response; Trach, tracheostomy; Tx, treatment; Vent, ventilator; Wks, weeks.

### Clinical course after IL-2 discontinuation

**Figure 1:**
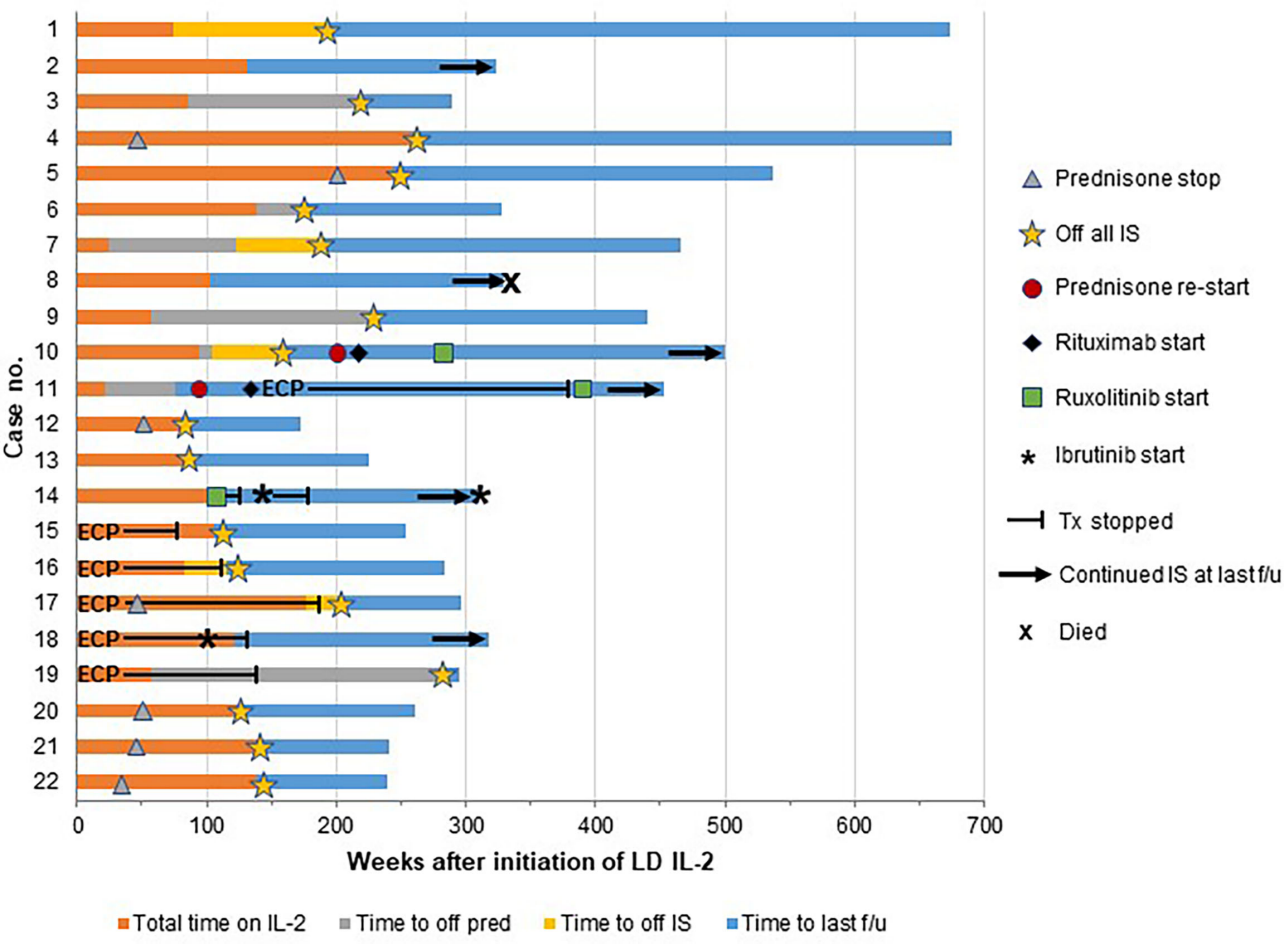
Clinical outcomes after IL-2 discontinuation. Each patient is represented by a horizontal bar with the time on low-dose IL-2 (LD IL-2) represented in orange and the time after IL-2 discontinuation represented in blue. The majority of the patients were able to discontinue all immunosuppression after stopping LD IL-2, but 3 patients (case numbers 10, 11, and 14) had progression of chronic GVHD requiring initiation of additional immunosuppression and 3 patients (case numbers 2, 8, and 18) continued on their prior immunosuppressive agents at the time of last follow up. Time to discontinuation of prednisone is represented as gray bars if prednisone was stopped after IL-2 discontinuation. If prednisone was weaned off during the IL-2 treatment course, the time of discontinuation is represented by a gray triangle. The time to discontinuation of all immunosuppression (IS) is represented as yellow bars and yellow stars. ECP, extracorporeal photopheresis; f/u, follow up; no., number; pred, prednisone; Tx, treatment.

After IL-2 discontinuation, patients had a median follow up time of 203 weeks (range, 92-599). One patient (C8) died from multisystem organ failure following a prolonged hospitalization for appendicitis 227 weeks after stopping LD IL-2. All of the remaining 21 patients were alive at the time of last follow up (**Figure 1**, **Table 2**). Fifteen patients (68%) weaned off prednisone at a median of 84 weeks (range, 35-280) following the start of LD IL-2. One patient (C11) experienced worsening of her vaginal cGVHD upon steroid cessation and was restarted on prednisone 16 weeks later. She subsequently received a course of rituximab and also received extracorporeal photopheresis (ECP) for 249 weeks. Due to persistent vaginal symptoms, ECP was stopped and ruxolitinib was started at 390 weeks after stopping LD IL-2. The patient remained on ruxolitinib at her last follow up 63 weeks later.

Seventeen patients (77%) were able to come off all cGVHD-directed systemic immunosuppression at a median of 156 weeks (range, 76-280) from the start of LD IL-2 therapy (**Table 2**). Sixteen of these patients remained off all immunosuppression without recurrence or progression of cGVHD at a median follow-up of 144 weeks after LD IL-2 discontinuation (range, 15-481) (**Figure 1**). One patient (C10) remained stable for 52 weeks after stopping LD IL-2 but then experienced progression of her skin and joint cGVHD involvement. She was given a prednisone pulse followed by a course of rituximab without significant improvement. She ultimately achieved a PR with ruxolitinib and remained continuously on ruxolitinib at last follow up (343 weeks after stopping LD IL-2).

Four additional patients remained on systemic immunosuppression after discontinuation of LD IL-2. Two of these patients (C2 and C8) had stable disease on low-dose prednisone combined with sirolimus or tacrolimus, respectively. Patient C8 subsequently died from complications related to appendicitis as mentioned above. One patient (C14) discontinued LD IL-2 due to plateaued response and immediately started on ruxolitinib while remaining on sirolimus in an attempt to deepen the response in his skin and joints. He discontinued ruxolitinib after 13 weeks due to lack of response. He was then started on ibrutinib and had some further improvement but had to stop after 41 weeks due to side effects. At his last follow up (206 weeks after stopping LD IL-2), he remained on sirolimus and restarted ibrutinib due to a flare of his skin cGVHD. Lastly, patient C18 had a relapse of CLL during week 121 of LD IL-2 therapy. Ibrutinib was started and LD IL-2 was stopped while continuing ECP and prednisone. Her skin cGVHD continued to improve and ECP was stopped 15 weeks later. She continued on low-dose prednisone and ibrutinib to control both her CLL and cGVHD at her last follow up (196 weeks after IL-2 discontinuation).

### T cell response after IL-2 discontinuation

**Figure 2:**
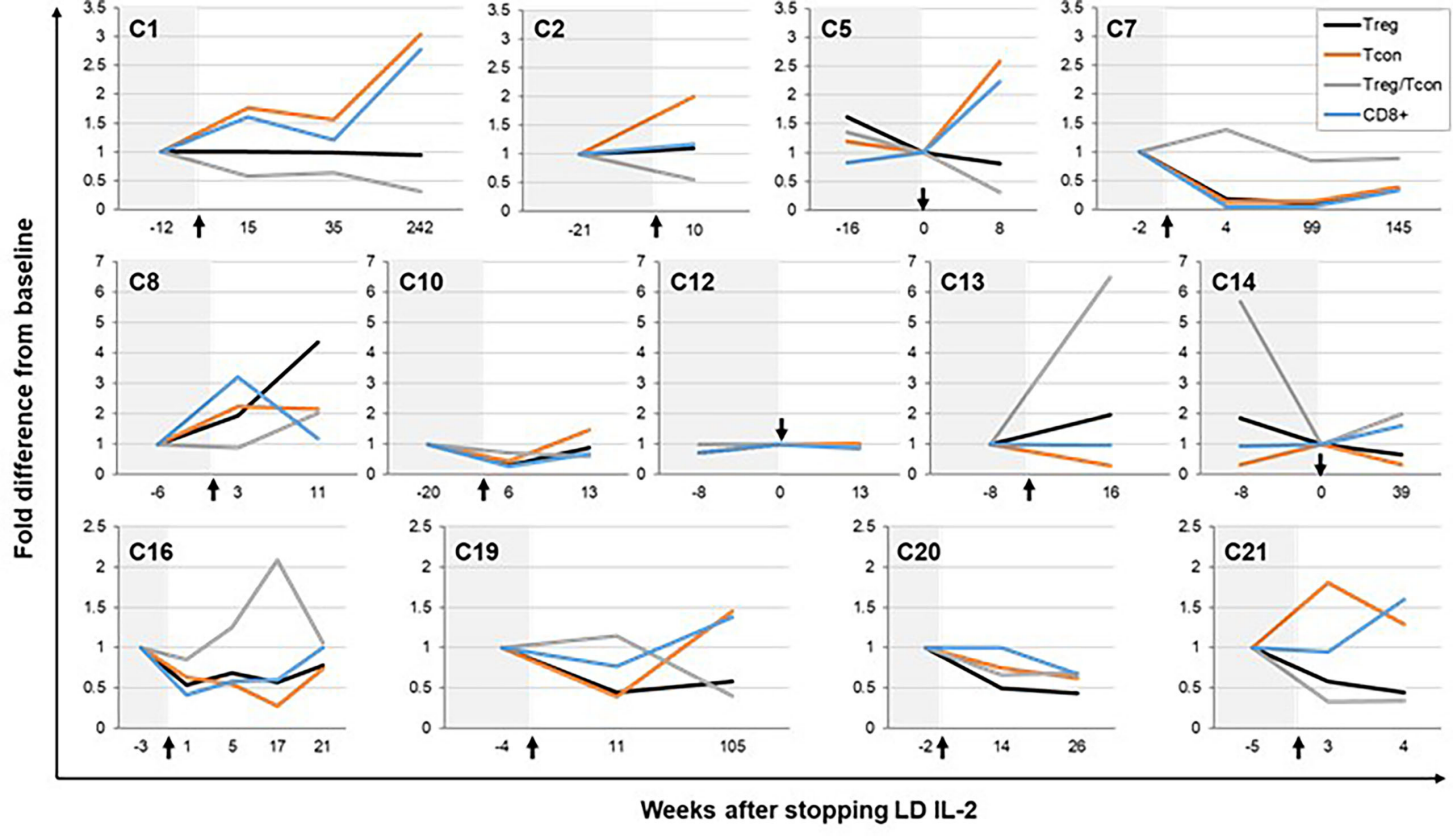
Immune responses after IL-2 discontinuation. Fold change from baseline in CD4+ regulatory T cell (Treg), CD4+ conventional T cell (Tcon), and CD8+ T cell numbers after stopping low-dose IL-2 therapy is shown for patients who had available immune cell data. Baseline was considered as the earliest time point prior to IL-2 discontinuation where immune cell data was obtained except for in 3 patients (C5, C12, and C14), who had data available on the day of IL-2 discontinuation (time point 0). Each graph represents data from an individual patient. Treg are represented by black lines, Tcon by orange lines, Treg/Tcon ratio by gray lines, and CD8+ T cells by blue lines. The time period prior to IL-2 discontinuation is shaded in light gray. Black arrows represent the time of IL-2 discontinuation.

We examined CD4+ Treg, CD4+ Tcon, and CD8+ T-cell numbers in peripheral blood of thirteen patients who had available blood samples before and after stopping LD IL-2 (**Figure 2**). There was a median of 2 post-IL-2 discontinuation time points (range, 1-4) collected from these patients. The median length of immune cell follow up after stopping IL-2 was 16 weeks (range, 4-242). During this time, Treg numbers decreased in 7 patients (54%), remained the same in 4 patients (31%), and increased in 2 patients (26%) (**Supplementary Table 1**). At the time of last follow up for all 13 patients, the median fold difference in absolute Treg number relative to the most recent available time point prior to IL-2 discontinuation was 0.81 (range, 0.34-4.4). When the follow up period is divided into intervals, the median fold difference in absolute Treg number compared with the most recent baseline prior to stopping IL-2 was 0.58 at 1 to 10 weeks, and 0.89 at 11-20 weeks after IL-2 discontinuation (**Supplementary Figure 1A**). Although not statistically significant, the median differences in Treg number after stopping IL-2 are higher than the difference between the baseline on LD IL-2 therapy and the Treg number prior to starting IL-2 (median 0.22-fold lower) (**Supplementary Figure 1A**). Thus, despite a decrease after stopping LD IL-2, Treg numbers appear to stabilize at a level higher than the pre-IL-2 baseline for the majority of patients (**Supplementary Figure 1B**). At the last follow up time point for all 13 patients, the Treg/Tcon ratio also decreased to a median of 0.7 times the value prior to stopping IL-2 (range, 0.3-6.5). For Tcon and CD8+ T cells, the median fold difference was 1.3 (range, 0.3-3) and 1.2 (range, 0.3-2.8), respectively.

**Figure 3:**
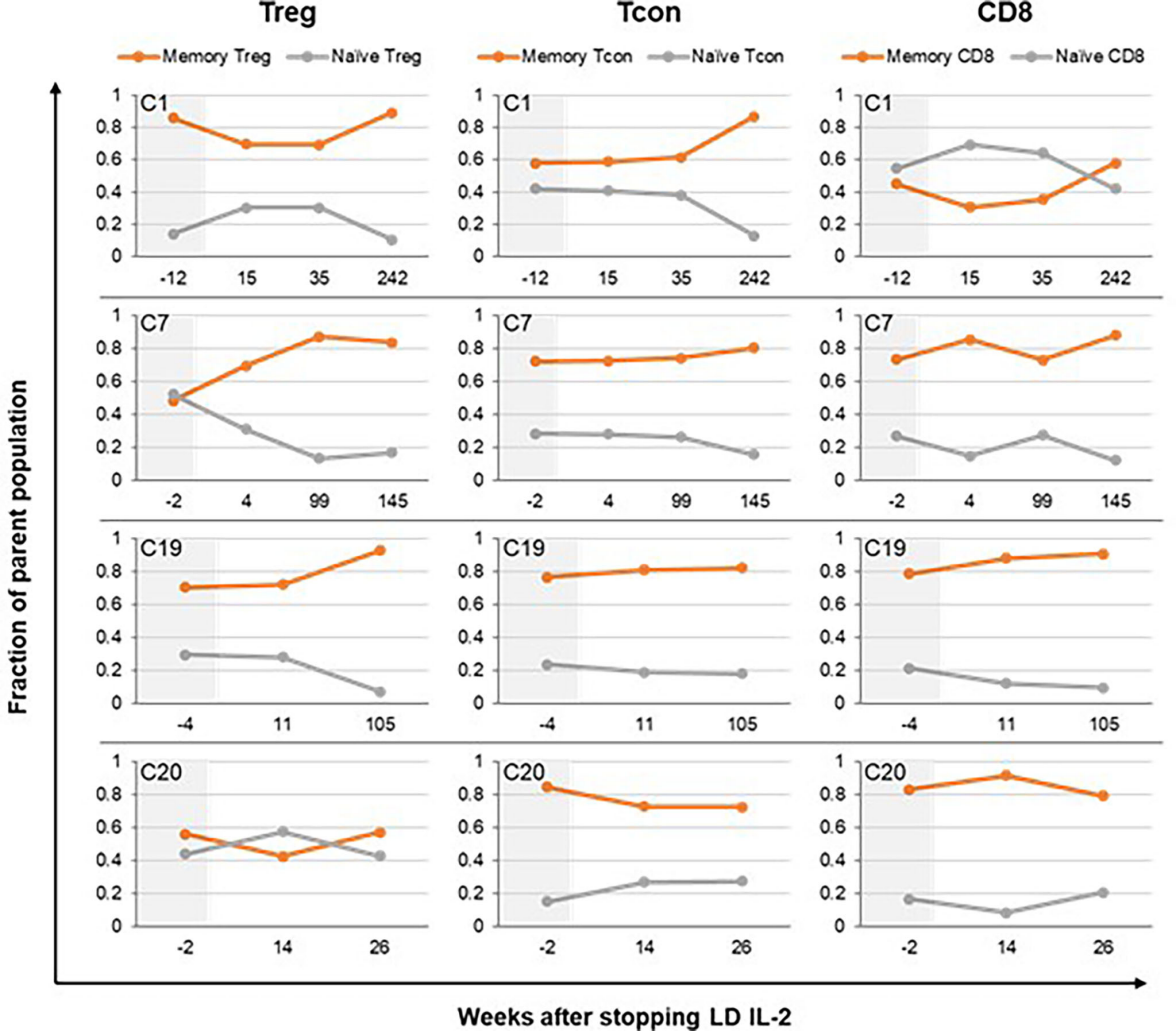
Impact of IL-2 discontinuation on naïve and memory T-cell compartments. Naïve and memory subsets within each of the T cell parent populations (CD4+ Treg, CD4+ Tcon, and CD8+ T cells) are shown. The fraction of naïve T cells within Treg, Tcon, and CD8+ T-cells over time is represented by orange lines. The fraction of memory T cells within the various T cell subtypes is represented by gray lines. The memory fraction includes effector memory, central memory, and TEMRA T cells. Patient C1, C7, and C19 had the longest follow up period with available immune cell data. Patient C20 is shown as representative data for patients with shorter follow up times. The time period prior to IL-2 discontinuation is shaded in light gray. Treg, CD4+ regulatory T cell; Tcon, CD4+ conventional T cell.

We also examined changes in naïve and memory subsets within the CD4+ Treg, CD4+ Tcon, and CD8+ T-cell populations following IL-2 discontinuation. For the three patients (C1, C7, C19) with the longest post-IL-2 follow-up, there was a trend toward decrease in the naïve fraction and increase in the memory fraction within all the T cell subtypes, but most pronounced within the Tregs (**Figure 3**). This is consistent with our prior observation that LD IL-2 leads to expansion of the naïve subset within Tregs and improves thymic output (18). Patients with shorter follow-up (less than 6 months) showed relative stability in the naïve and memory fractions within all the T cell subtypes. Representative data is shown from patient C20 in **Figure 3** and data for all patients is shown in **Supplementary Figure 2**.

## Discussion

Over the past 15 years, we have established that daily LD IL-2 causes a rapid preferential expansion of CD4+ Tregs and subsequently leads to clinical improvement in patients with refractory cGVHD. Reversal of cGVHD manifestations with LD IL-2 can be observed in patients who have failed multiple prior lines of therapy. Although LD IL-2 can only be given as a subcutaneous injection, a continuous daily regimen was well-tolerated and 70 of 120 (58%) evaluable patients with clinical improvement remained on extended therapy. Across our center’s 5 clinical trials, 35 of the 70 (50%) patients had deeper responses during the extended treatment phase. However, the optimal duration of therapy is unknown, and some patients remained on study for years due to a fear of cGVHD recurrence or progression after IL-2 discontinuation. Of the 22 patients with positive clinical responses in this report, the median duration of LD IL-2 therapy was 103 weeks including two patients who continued daily injections for nearly 5 years. To examine the durability of clinical and immune response after a prolonged course of LD IL-2, we identified 22 patients who stopped LD IL-2 therapy after achieving maximal benefit.

We found that 17 of the 22 patients (77%) were able to wean off all additional systemic immunosuppression during or after their LD IL-2 course. Sixteen patients remained off systemic immunosuppression with stable residual disease at a median follow-up time of 144 weeks. One patient (C10) was stable for 1 year after weaning off all immunosuppression, but then experienced worsening of cGVHD that required re-initiation of systemic immunosuppression. Five additional patients (C2, C8, C11, C14, C18) discontinued LD IL-2 due to plateaued response but continued other systemic therapies due to persistent cGVHD symptoms. Due to the small sample size, it is not possible to identify correlates of durable response to LD IL-2, but we noted that all 6 patients who required continued cGVHD-directed immunosuppressive therapy after stopping LD IL-2 had persistent skin involvement. This is consistent with the observation that sclerodermatous skin is difficult to reverse and is one of the most debilitating cGVHD manifestations (19).

When we examined Treg numbers after LD IL-2 discontinuation in 13 patients with available immune cell data, there was no consistent pattern that correlated with clinical outcomes. Some patients who weaned off all immunosuppression with sustained remission of their cGVHD had Treg numbers that decreased over time (C20, C21) whereas some patients who remained on immunosuppression had stable or increased Treg numbers (C2, C8, C10). Similarly, although expansion of the naïve fraction within the CD4+ T cell population suggests thymopoiesis and is required for immune tolerance, we observed a decrease in the naïve fraction within CD4+ Treg and Tcon in several patients (C1, C7, C19, C21) whose cGVHD remained quiescent off all immunosuppression post-IL-2. We have previously shown that overall Treg expansion and Treg phenotypes are similar between LD IL-2 responders and non-responders (18). A more recent organ-specific analysis of clinical and immune correlates of response to LD IL-2 in adult patients identify some early predictors of response such as higher Treg/Tcon ratio and lower terminal effector memory T cell counts within the first 4 weeks of therapy (17), but markers of continued response or disease progression during extended therapy have not been identified. Thus, predictive biomarkers to guide treatment duration for LD IL-2 and other second-line therapies remains an area of unmet need.

Consistent with previous observations, the course of cGVHD and its treatment in our cohort was prolonged (20, 21). At last follow-up, patients in this cohort were a median of 8.8 years post-transplant (range, 4.8-17.2), yet some patients still had not achieved immune tolerance and were having persistent cGVHD symptoms. Despite the requirement for daily injections, LD IL-2 is well-tolerated and has efficacy in patients with SR-cGVHD. All 22 patients in this cohort, including 2 pediatric patients, were highly motivated to remain on therapy due to improvement of their disease. Many of these patients continued therapy despite plateaued responses due to fear that their cGVHD manifestations would recur. Although we have shown that the clinical and immunologic effects of LD IL-2 can be sustained after stopping extended treatment, the optimal duration of therapy remains unknown. While it is fortunate that the number of second-line treatment options for SR-cGVHD has expanded with the recent FDA approval of ibrutinib, ruxolitinib, and belumosudil, the optimal treatment course for these agents is also undefined. Thus, biomarkers for durability of response to LD IL-2 or other second-line therapies are important tools that could help to avoid unnecessary prolonged immunosuppression. Our observations also underscore a need to focus efforts on a better understanding of immune reconstitution after allogeneic hematopoietic stem cell transplantation and approaches to enhancing earlier immune tolerance as an approach to preventing cGVHD.

## Data availability statement

The original contributions presented in the study are included in the article/**Supplementary Material**. Further inquiries can be directed to the corresponding author.

## Ethics statement

This study was reviewed and approved by Dana-Farber/Harvard Cancer Center Institutional Review Board. Written informed consent to participate in this study was provided by the participants’ legal guardian/next of kin.

## Author contributions

JW, JR and JK conceived and designed the study. VD, HK, PS, CR and JW collected and assembled data. All authors contributed to the article and approved the submitted version.

## Funding

This work was supported by NIH grants P01CA229092, P01CA142106 and P30CA006516.

## Acknowledgments

The authors thank the patients who participated in these trials. The authors thank the Pasquarello Tissue Bank in Hematologic Malignancies for prospective collection and processing of serial blood samples.

## Conflict of interest

JK receives research support from BMS, Miltenyi Biotec, Novartis, Clinigen, Regeneron; serves on Scientific Advisory Boards for Therakos, Cugene, Biolojic Design; consults for Amgen, Gentibio, Equillium, EMD Serono/Merck, Moderna. JR receives research funding from Kite/Gilead and Novartis, serves on Data Safety Monitoring Committees for AvroBio and Scientific Advisory Boards for Akron Biotech, Clade Therapeutics, Draper Laboratories, Garuda Therapeutics, LifeVault Bio, Novartis, Rheos Medicines, Smart Immune, Talaris Therapeutics and TScan Therapeutics.

The remaining authors declare that the research was conducted in the absence of any commercial or financial relationships that could be construed as a potential conflict of interest.

The reviewer JM declared a past co-authorship with the author JK to the handling editor.

## Publisher’s note

All claims expressed in this article are solely those of the authors and do not necessarily represent those of their affiliated organizations, or those of the publisher, the editors and the reviewers. Any product that may be evaluated in this article, or claim that may be made by its manufacturer, is not guaranteed or endorsed by the publisher.

## Supplementary material

Supplementary Figure 1 Absolute Treg counts before and after IL-2 discontinuation. **(A)** Fold change from baseline prior to LD IL-2 discontinuation. Values at all time points, including the Treg count prior to starting LD IL-2 therapy (Pre IL-2) were normalized to the absolute Treg count for each patient at the last time point prior to stopping LD IL-2. **(B)** Absolute Treg count trends over time are shown for each individual patient. * p < 0.05.

Supplementary Figure 2 Impact of IL-2 discontinuation on naïve and memory T-cell compartments. The fraction of naïve T cells within Treg, Tcon, and CD8+ T-cells over time is represented by orange lines. The fraction of memory T cells within the various T cell subtypes is represented by gray lines. The memory fraction includes effector memory, central memory, and TEMRA T cells. Patients’ data is displayed in individual rows and patients are designated by their case numbers. Treg, CD4+ regulatory T cell; Tcon, CD4+ conventional T cell.
