# Supplementary figures and images for: Durability of clinical and immunologic responses to extended low-dose interleukin-2 therapy in patients with refractory chronic graft-versus-host disease
Source: Front Immunol. 2022 Sep 14;13:954966. doi: 10.3389/fimmu.2022.954966 (PMC9515381; doi:10.3389/fimmu.2022.954966)

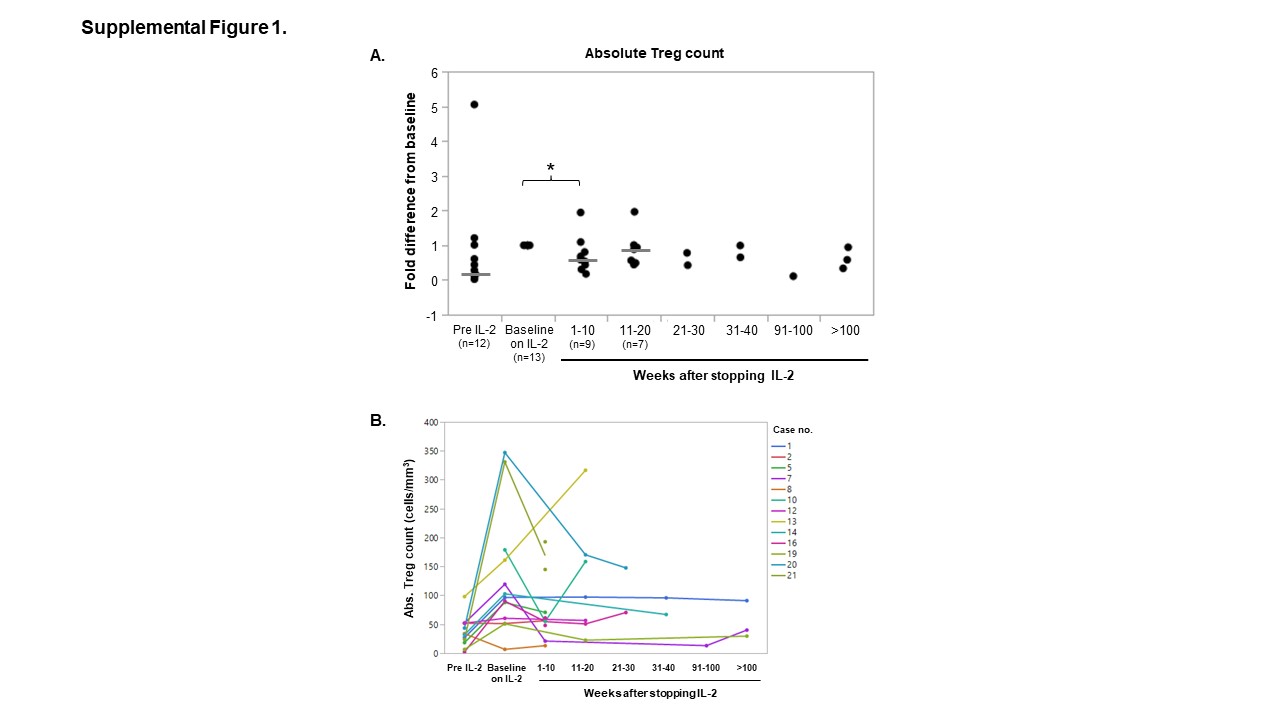

Supplement: Supplementary Figure 1 — Absolute Treg counts before and after IL-2 discontinuation. (A) Fold change from baseline prior to LD IL-2 discontinuation. Values at all time points, including the Treg count prior to starting LD IL-2 therapy (Pre IL-2) were normalized to the absolute Treg count for each patient at the last time point prior to stopping LD IL-2. (B) Absolute Treg count trends over time are shown for each individual patient. * p < 0.05. [file Image_1.jpeg]

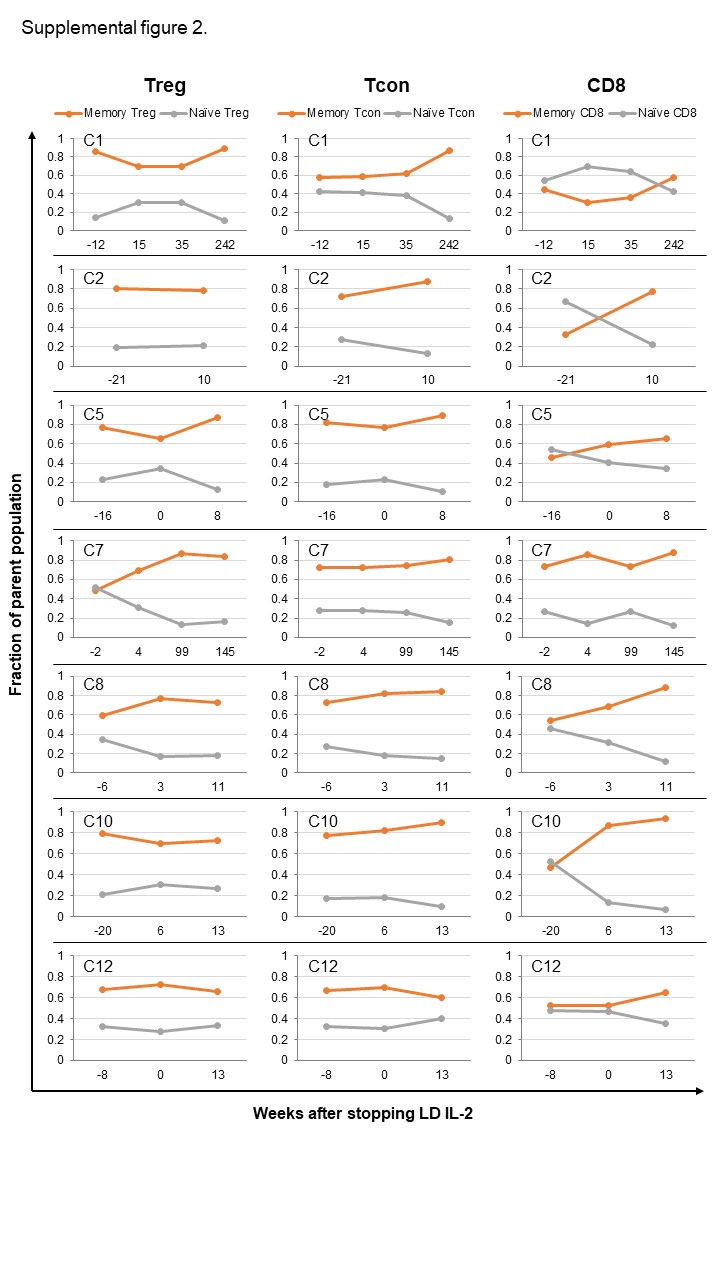

Supplement: Supplementary Figure 2 — Impact of IL-2 discontinuation on naïve and memory T-cell compartments. The fraction of naïve T cells within Treg, Tcon, and CD8+ T-cells over time is represented by orange lines. The fraction of memory T cells within the various T cell subtypes is represented by gray lines. The memory fraction includes effector memory, central memory, and TEMRA T cells. Patients’ data is displayed in individual rows and patients are designated by their case numbers. Treg, CD4+ regulatory T cell; Tcon, CD4+ conventional T cell. [file Image_2.jpeg]

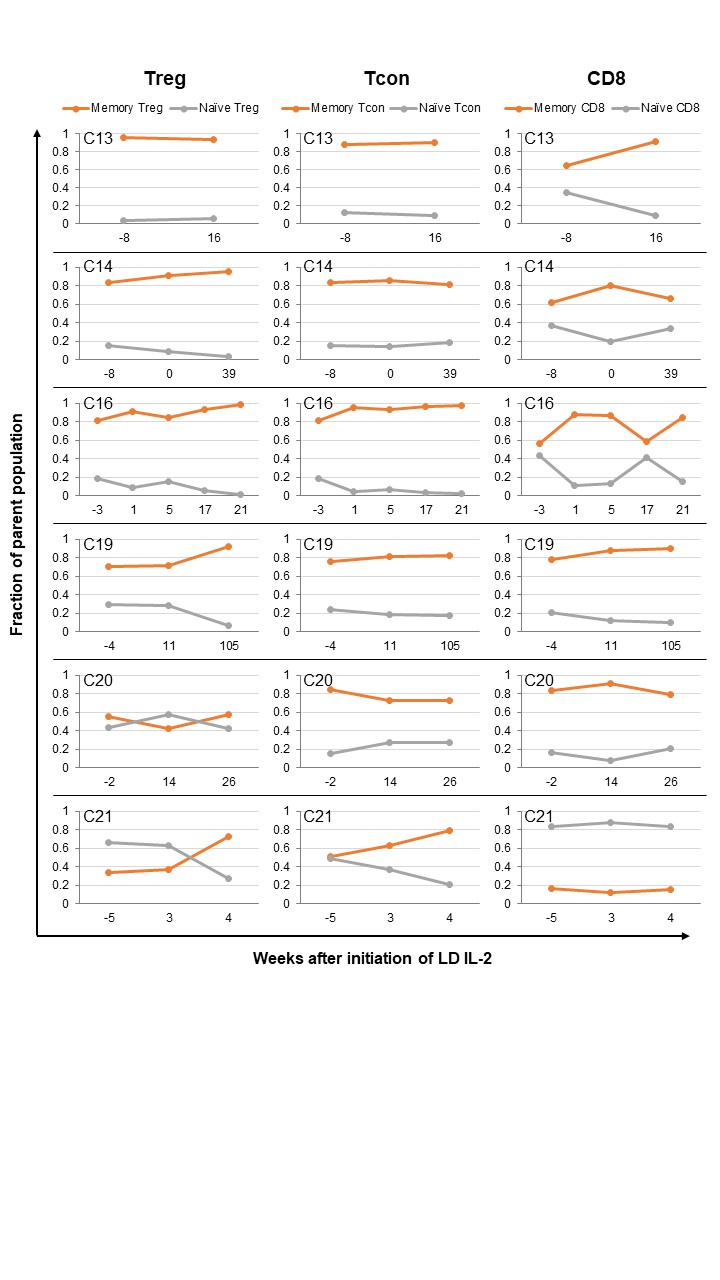

Supplement: Supplementary file 3 [file Image_3.jpeg]
