# Supplementary material for: Durability of clinical and immunologic responses to extended low-dose interleukin-2 therapy in patients with refractory chronic graft-versus-host disease
Source: Front Immunol. 2022 Sep 14;13:954966. doi: 10.3389/fimmu.2022.954966 (PMC9515381; doi:10.3389/fimmu.2022.954966)
Supplement: Supplementary file 4 [file Table_1.docx]

Supplemental Table 1. Absolute Treg count before and after stopping LD IL-2

|  |  | **Abs. Treg count (cells/mm^3^)** | |  |  |
| --- | --- | --- | --- | --- | --- |
| **Case no.** | **Pre-treatment** | **End of LD IL-2** | **Last follow up** | **TP last follow up (Wks)** | **cGVHD and IS status at last follow up** |
| 1 | 27 | 96.5 | 91 | 242 | Improving subcutaneous fibrosis; off all IS |
| 2 | 52 | 51.4 | 56.2 | 10 | Stable thickened skin; on sirolimus and LD prednisone |
| 5 | 18.4 | 87.8 | 70.7 | 8 | No active disease; off all IS |
| 7 | 52.2 | 119.4 | 40.1 | 145 | Minimal cGVHD of the eyes; off all IS |
| 8 | 33.8 | 6.7 | 29.3 | 11 | Progressive lung and active skin cGVHD; on tacrolimus and prednisone |
| 10 | 178.9 | 178.9 | 158.6 | 13 | Continued sclerodermatous skin; on ruxolitinib and LD prednisone |
| 12 | 52 | 60.4 | 56.7 | 13 | No active disease; off all IS |
| 13 | 98 | 161.1 | 316.5 | 16 | Persistent mild skin and JMF involvement; off all IS |
| 14 | 32 | 102.7 | 67 | 39 | Persistent severe cGVHD of skin and JMF; on sirolimus and ibrutinib |
| 16 | 2.2 | 90.2 | 70.4 | 21 | Residual scleroderma; off all IS |
| 19 | 6.6 | 50.9 | 29.6 | 105 | No active disease; off all IS |
| 20 | 43.6 | 347.1 | 147.7 | 26 | Persistent eye involvement; off all IS |
| 21 | 24 | 330.8 | 144.9 | 4 | Residual hyperpigmented skin; off all IS |

Abs.. absolute; cGVHD, chronic graft-versus-host disease; IS, immunosuppression; JMF, joint/muscle/fascia; LD, low-dose; TP, time point; Wks, weeks
